# Self‐Responsive Plastron Maintenance on a Superhydrophobic Mesh via Locally Integrated Electrolysis

**DOI:** 10.1002/advs.78069

**Published:** 2026-10-08

**Authors:** Jiaming Wang, Zhen Zhang, Yuhong Liu

**Affiliations:** ^1^ Key Laboratory of Tribology in Advanced Equipment Tsinghua University Beijing China

**Keywords:** anti‐scaling, electrolysis, plastron, self‐responsive, superhydrophobic

## Abstract

Underwater superhydrophobic surfaces can retain an interfacial gas layer (known as plastron), enabling functions such as drag reduction and antifouling. However, the plastron suffers from inevitable instability under hydrostatic pressure and flow‐induced shear. Current electrolysis‐based replenishment strategies are often hindered by complex electrode configurations, gas leakage, and a lack of autonomous feedback. Herein, we present a self‐responsive superhydrophobic mesh with locally integrated electrodes (SHM‐E) to achieve intelligent plastron maintenance. By coupling a compact local Pt anode (occupying only 1/16 of the functional surface area) with the capillary‐driven gas spreading of the mesh, the system realizes an autonomous “damage–repair–dormancy” cycle. This configuration enables rapid plastron restoration (<60 s) and sustains near‐perfect coverage for over 16 days with minimal power consumption (static current <1 mA), significantly outperforming non‐electrolysis controls. Consequently, the stabilized plastron effectively isolates the substrate, reducing scale deposition by 93.8% compared to stainless steel. Overall, this study demonstrates an unattended, low‐power, self‐responsive plastron repair strategy that simplifies electrolysis‐based gas replenishment, providing a practical route toward durable underwater functional surfaces.

## Graphical abstract

This work presents a self‐switchable SHM‐E surface that enables rapid, autonomous plastron self‐repair and long‐term stability. Plastron loss triggers circuit closure and localized electrolysis, while capillary spreading rapidly restores a continuous plastron and self‐terminates the current. Complete recovery is achieved within 60 s (10 V voltage supply), with >95% coverage for 16 days at 5 V and 93.8% less CaCO_3_ deposition.

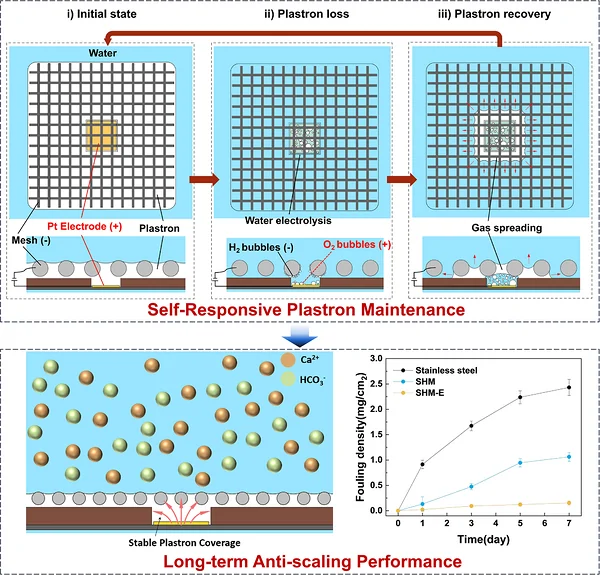

## 1 Introduction

The interfacial gas layer (plastron) retained at the solid–liquid interface is the key carrier for the unique functions of underwater superhydrophobic surfaces [1, 2, 3, 4, 5, 6]. In drag reduction, the plastron can reduce interfacial friction via slip effects, thereby lowering energy consumption in ships and pipelines [7, 8, 9, 10, 11]. In antifouling and anti‐scaling, the plastron acts as a physical barrier that prevents direct contact between microorganisms/scale crystals and the substrate, suppressing bio‐adhesion and scale growth at the source [12, 13, 14, 15]. In addition, the plastron can provide a stable gas–liquid–solid triple‐phase interface for underwater reactions, showing promise in energy conversion and environmental remediation [16, 17, 18, 19]. Previous work has demonstrated that superhydrophobic mesh surfaces can achieve efficient gas spreading and stable plastron retention through capillary effects, establishing a structural basis for plastron‐enabled applications [20, 21, 22].

Despite its importance, the plastron is thermodynamically metastable underwater [23, 24, 25]. Increasing hydrostatic pressure with depth, shear induced by fluid flow, and spontaneous dissolution of gas into water can compress, detach, or fully deplete the plastron, triggering the Cassie–Wenzel wetting transition [26, 27, 28, 29]. This transition is typically irreversible; once it occurs, the surface loses its special interfacial functions [30]. To improve plastron stability, passive protection strategies based on hierarchical roughness, re‐entrant structures, pressure balancing, and hydrophilic pinning have been developed to enhance plastron robustness without external energy input [31, 32, 33, 34]. In contrast, active replenishment strategies restore depleted plastrons through in situ or externally supplied gas, including direct gas injection, chemical gas generation, and water electrolysis [35, 36, 37, 38, 39, 40, 41, 42, 43]. Among them, in situ gas generation by water electrolysis is considered attractive because the gas source is sustainable and can be readily controlled by electrical input [38, 39, 40, 41, 42, 43].

Nevertheless, current electrolysis‐based plastron replenishment approaches still face several challenges. First, most systems employ spatially separated electrodes (with the cathode on the superhydrophobic surface and the anode suspended in the solution), resulting in redundant architectures and inefficient utilization of anode‐generated gas, thereby lowering energy efficiency [39, 40]. Second, to achieve uniform recovery, some designs require full‐area electrodes covering the superhydrophobic surface, increasing fabrication cost and potentially compromising the micro/nanostructures responsible for plastron retention and interfacial stability [40, 42]. Third, switching electrolysis on and off often relies on external sensing/feedback control to monitor the plastron state, which increases system complexity and makes reliable, long‐term, low‐power operation difficult [43]. Although several studies have exploited water infiltration following plastron loss to autonomously trigger gas‐generating reactions, these wetting‐responsive systems still commonly rely on spatially separated electrodes or active layers covering most or all of the functional surface [36, 38, 40]. Therefore, developing a highly integrated replenishment strategy that efficiently utilizes generated gas and can autonomously trigger and terminate in response to plastron status is essential for practical underwater superhydrophobic interfaces.

To address these limitations, we developed a superhydrophobic mesh electrolysis surface (SHM‐E) by synergizing the capillary‐driven gas spreading capability of the mesh with a locally integrated electrode configuration. The SHM‐E features a compact, integrated architecture: the superhydrophobic mesh serves as the cathode, while a localized Pt anode is positioned centrally (occupying only 1/16 of the total area). This design eliminates the need for an external suspended counter electrode and minimizes structural redundancy. Leveraging capillary action, gas generated at the localized anode spreads rapidly across the mesh surface, thereby avoiding the complexity of full‐area electrodes while reducing fabrication costs and energy consumption. Crucially, the SHM‐E enables an autonomous response to wetting where the circuit remains dormant when the plastron is intact but spontaneously activates upon water infiltration. As the generated gas expands and expels the water, the circuit is disconnected, automatically terminating the electrolysis. This enables an intelligent, unattended “damage–repair–dormancy” cycle.

## 2 Results and Discussion

### 2.1 Fabrication Process and Characterization of the SHM‐E Surfaces

The fabrication process of SHM‐E is shown in Figure 1a. Stainless‐steel mesh was employed as the substrate because its intrinsic micrometer‐scale texture facilitates the construction of hierarchical dual‐scale roughness. Briefly, a commercial superhydrophobic coating (NeverWet), which consists of a solvent and hydrophobic nanoparticles, was spray‐coated onto the stainless‐steel mesh to obtain a superhydrophobic mesh. The mesh, substrate, and local electrode were then integrated using foam tape (Figure 1a). A Pt plate anode was positioned at the center of the substrate, while the superhydrophobic stainless‐steel mesh served as the cathode. The Pt anode area (1 cm × 1 cm) corresponded to 1/16 of the functional surface area (4 cm × 4 cm). A cavity with a height of ∼0.8 mm was formed between the two electrodes by the foam spacer. This cavity enables temporary gas storage and lateral transport and, importantly, directly couples “water ingress” with circuit connection/disconnection, providing a structural basis for self‐responsive electrolysis‐driven gas replenishment.

**FIGURE 1:**
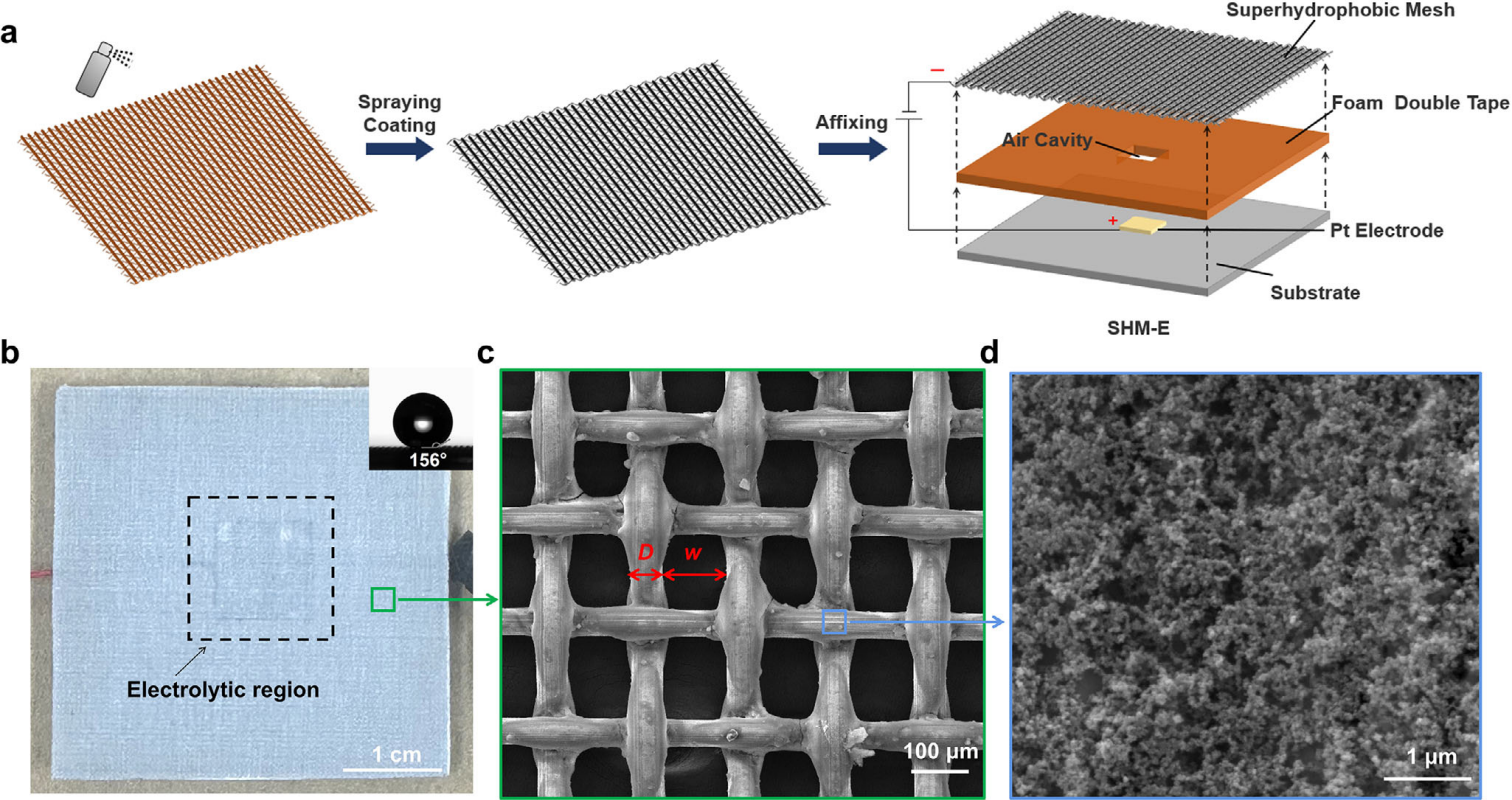
Fabrication process and characterization of SHM‐E. (a) Two‐step fabrication process of the superhydrophobic mesh electrolysis surface (SHM‐E) and the exploded schematic of its structure. (b) Optical image of the as‐prepared SHM‐E. (c, d) Scanning electron micrographs of the hierarchical micro‐nano structure on SHM‐E at different magnifications.

An optical photograph of the as‐prepared sample is shown in Figure 1b, where the top surface is covered by the superhydrophobic mesh. The mesh with a mesh number of 150 has a wire diameter (*D*) of ∼63 µm and a pore size (*W*) of ∼110 µm. SEM images reveal a typical hierarchical micro/nanostructure: a micrometer‐scale mesh skeleton (Figure 1c) decorated with nanoparticles (Figure 1d) with sizes of ∼20–40 nm. Moreover, the surface topography and chemical characteristics of the used NeverWet coating have been well documented in earlier reports [22, 44]. The nanoscale features of the coating are essential for boosting hydrophobicity by retaining air within nanocavities, thereby generating a stable solid–air–liquid composite interface and effectively increasing the apparent water repellency [45]. The combined micro‐scale framework and nano‐scale roughness form a multiscale texture, yielding superhydrophobicity with a water contact angle of 156.2° ± 3.5° and a sliding angle of 1.5° ± 0.25°. Overall, the electrodes are integrated on the same surface with a compact layout, which is beneficial for reducing system volume and structural complexity.

### 2.2 Electrolysis‐Enabled Plastron Self‐Recovery

To evaluate the plastron self‐recovery capability of SHM‐E, the complete “removal–recovery” process was recorded. The relative reflectivity was used to quantify plastron coverage, and the current was monitored simultaneously during recovery (Figure 2a,b). The porous mesh can retain a micrometer‐thick plastron within the openings, forming a Cassie‐Baxter wetting state [46]. The plastron induces specular reflection and leads to an apparent brightness contrast [21, 43]. When the incident light is fully reflected away from the camera, the plastron‐covered surface appears dark, whereas after plastron loss the surface becomes bright due to diffuse reflection entering the camera. Therefore, the relative reflectivity can be used to indicate plastron coverage.

**FIGURE 2:**
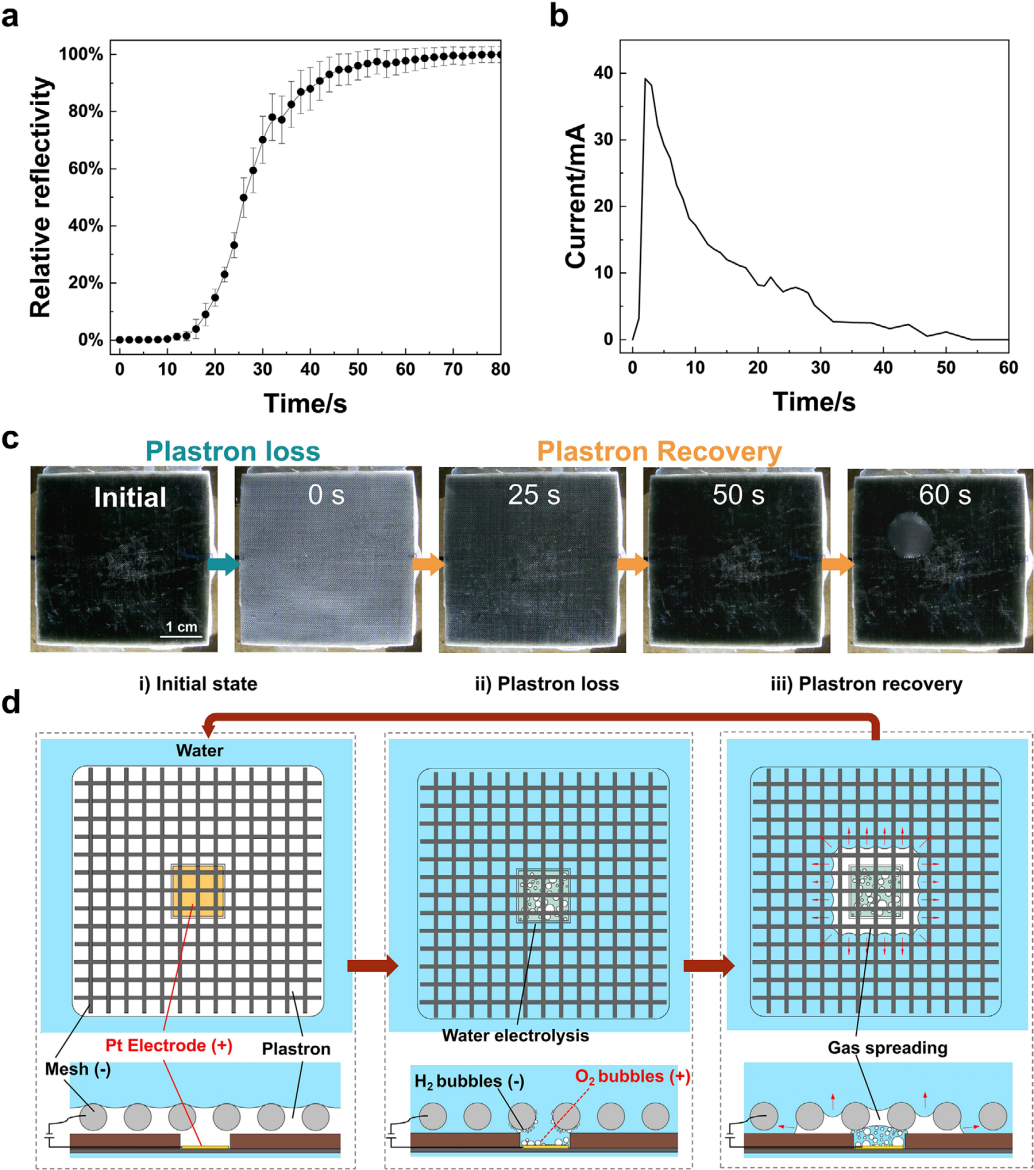
Rapid plastron self‐recovery of SHM‐E. (a) Variation of relative reflectivity with time during the plastron self‐recovery process. (b) Variation of current with time during the plastron self‐recovery process. (c) Optical images of plastron recovery on wetted SHM‐E. (d) Schematic of the plastron self‐recovery mechanism: Water infiltrates the central cavity, enabling circuit conduction and electrolysis of water; the generated gas spreads across the surface to restore the plastron, which re‐isolates the aqueous phase and terminates electrolysis automatically. The stainless‐steel mesh serves as the cathode (−), and the central Pt plate serves as the anode (+).

With the SHM‐E connected to a 10 V constant‐voltage supply, the surface initially exhibited a dark appearance with a relative reflectivity defined as 100% (Figure 2c). In this dormant state, the trapped plastron acted as an insulating barrier, effectively isolating the electrodes and preventing electrolysis. Upon mechanical removal of the gas layer by a pipette (*t* = 0 s), the surface transitioned to a bright, wetted state with the reflectivity dropping below 5%, which immediately closed the circuit and triggered the electrolytic reaction. Subsequently, a rapid recovery of plastron coverage was observed; the relative reflectivity surged to 96.5% within 50 s, indicating that the gas coverage had virtually returned to its initial level. Following this rapid restoration, while the reflectivity plateaued, a localized gas bulge formed near the central Pt anode, suggesting a brief continuation of electrolysis as residual water was expelled. By approximately 60 s, the bulge ceased to grow, marking the complete re‐establishment of electrode insulation and the autonomous termination of the process. Based on the recovery volume, the average gas generation rate during the first 50 s was calculated to be 4.4 µL/s (see Supporting Information for calculation details).

The plastron coverage correlates well with the current response (Figure 2b). At the early stage of recovery, as water infiltrated the cavity and electrolysis initiated, the current rapidly increased to 40 mA in approximately 3 s. As electrolysis proceeded, the generated gas spread and the plastron coverage increased; meanwhile, the water content in the cavity decreased, weakening ionic conduction and causing the current to gradually decline. By *t* = 50 s, when the plastron nearly fully covered the surface, the current decreased to 1.15 mA, suggesting that a small amount of water remained in the cavity and electrolysis continued, producing excess gas and forming the bulged bubble. By *t* = 60 s, as water was expelled and consumed during electrolysis, the current dropped to 0 mA, and the bubble ceased to grow, marking completion of recovery. These results confirm that SHM‐E enables an automatic switch between repair and stop. The present SHM‐E employs electrolysis as an active gas‐replenishment mechanism but operates in a self‐responsive manner, because wetting‐induced water ingress automatically activates electrolysis, whereas plastron restoration interrupts the conductive pathway and returns the system to a dormant state.

The self‐recovery follows a closed‐loop mechanism (Figure 2d): plastron damage → water ingress and circuit conduction → localized electrolysis‐driven gas generation → interfacial gas spreading and plastron restoration → water expulsion and circuit disconnection. Initially, the superhydrophobic mesh remains in the Cassie state with a continuous plastron, and the cavity is predominantly gas‐filled. Without a stable aqueous conductive pathway between cathode and anode, the circuit remains open (current ≈ 0), and electrolysis does not occur. Once the plastron is locally damaged, water enters the cavity through the mesh pores and forms an ion‐conducting path between electrodes. Upon applying a voltage, hydrogen evolution occurs at electrolyte‐accessible sites on the mesh cathode (2H_2_O + 2e^−^ → H_2_↑ + 2OH^−^), while oxygen evolution occurs at the Pt anode (4OH^−^ → O_2_↑ + 2H_2_O + 4e^−^). The generated gas spreads laterally along the solid–liquid interface driven by capillary action and interfacial spreading on the superhydrophobic mesh, gradually displacing the aqueous phase in the cavity and restoring a continuous plastron. As the gas progressively occupies the mesh–cavity structure, the water‐contacted regions connecting the two electrodes decrease until the continuous ionic pathway is interrupted. After the aqueous phase is expelled/isolated, the circuit becomes open again and electrolysis terminates automatically. Thus, the restored plastron acts as an intrinsic insulating barrier that terminates electrolysis even though the external voltage remains applied, returning the system to its dormant state.

It is also noted that the woven mesh structure cannot perfectly conform to the intermediate foam spacer; thus, small gaps exist between adjacent pores, allowing gas exchange and transport between neighboring pores, which facilitates gas spreading from the center toward the periphery [22]. In addition, due to the nanoscale roughness, even when the micrometer‐scale plastron in the pores is lost, the nanoparticles may retain nanoscale gas pockets (a nano‐Cassie state), which can promote gas spreading and facilitate plastron restoration. The residual nano‐Cassie state does not necessarily imply the persistence of a continuous electrically insulating gas film over the entire mesh. Following loss of the micrometer‐scale plastron, residual nanoscale gas pockets may coexist with locally wetted regions of the mesh, potentially allowing an aqueous conductive pathway to form between the electrodes without complete wetting of the nanostructure. The immediate current increase after plastron removal confirms conduction in the device and is consistent with this interpretation. Meanwhile, the residual nanoscale gas pockets can serve as preferential sites for gas nucleation and lateral spreading during recovery [47]. The different responses of the micro‐ and nanoscale gas domains originate from their distinct characteristic dimensions. The optically observable plastron is retained mainly within the approximately 110 µm mesh openings and the underlying cavity, and can be displaced when pipette aspiration produces a local pressure disturbance exceeding the corresponding capillary barrier. By contrast, nanoscale gas pockets confined within the nanoparticle coating encounter a much higher capillary barrier against liquid penetration because of their smaller characteristic dimensions and may therefore remain after removal of the micrometer‐scale plastron, as discussed in our previous work [21]. Accordingly, disappearance of the macroscopically continuous and reflective plastron does not imply complete wetting of every nanoscale cavity.

A control sample (hydrophilic mesh with electrolysis) exhibits markedly different gas evolution and electrical behavior. Due to the absence of superhydrophobicity, electrolysis‐generated gas cannot spread into a continuous plastron and instead forms discrete bubbles that rise and escape under buoyancy, preventing plastron recovery (Figure 3a). Correspondingly, because no insulating plastron forms to interrupt the circuit, the current rapidly increases after power is applied and remains around 30 mA (Figure 3b), indicating continuous electrolysis without self‐termination. This comparison highlights that the gas‐spreading capability of the superhydrophobic mesh is essential for “localized gas generation–global plastron recovery”, while the cavity design couples the plastron state with circuit conduction, enabling demand‐triggered self‐responsive repair.

**FIGURE 3:**
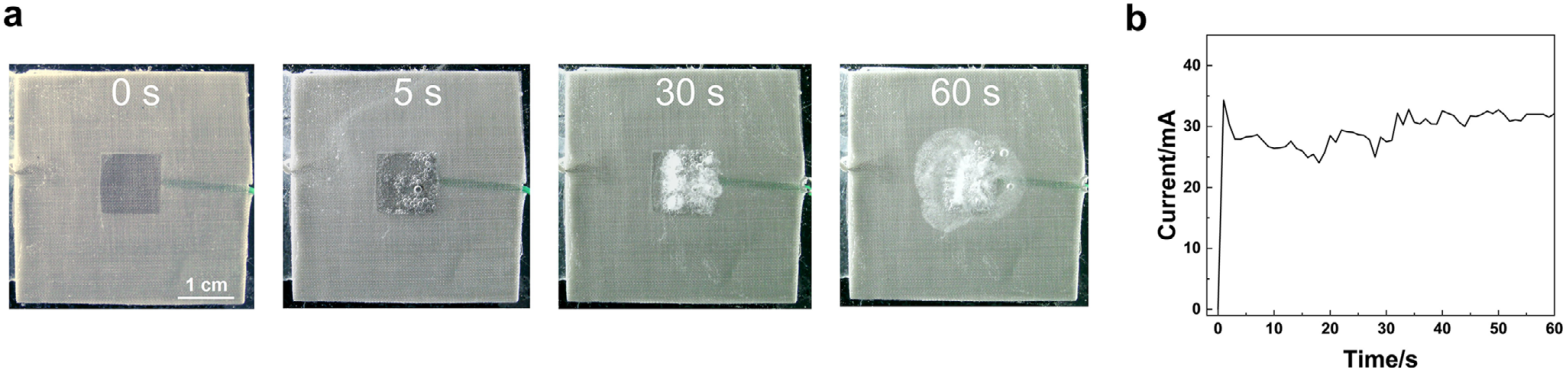
Electrolytic gas production process of mesh surfaces without superhydrophobic modification. (a) Optical images of the electrolysis process on mesh surfaces without superhydrophobic modification. (b) Variation of current with time during the electrolysis process.

### 2.3 Effect of Voltage on Plastron Recovery Efficiency

The applied voltage directly affects the gas generation rate and thus the timescale of plastron spreading and recovery. We evaluated plastron recovery of SHM‐E under different voltages (Figure 4a). Under a constant applied voltage of 5 V, the gas generation was moderate, leading to a gradual restoration process. To quantify the recovery efficiency, we defined *t*_90%_ as the duration required to attain 90% relative reflectivity. *t*_90%_ was measured to be 166 s when the applied voltage was 5 V. Increasing the voltage to 10 V significantly accelerated the kinetics, shortening *t*_90%_ to 42 s. Further elevating the potential to 15 V reduced *t*_90%_ to ∼20 s. At higher voltages of 20, 25, and 30 V, *t*_90%_ decreased to 9.5, 8.6, and 7.0 s, respectively (Figure 4b), indicating a diminishing return in recovery speed with increasing voltage.

**FIGURE 4:**
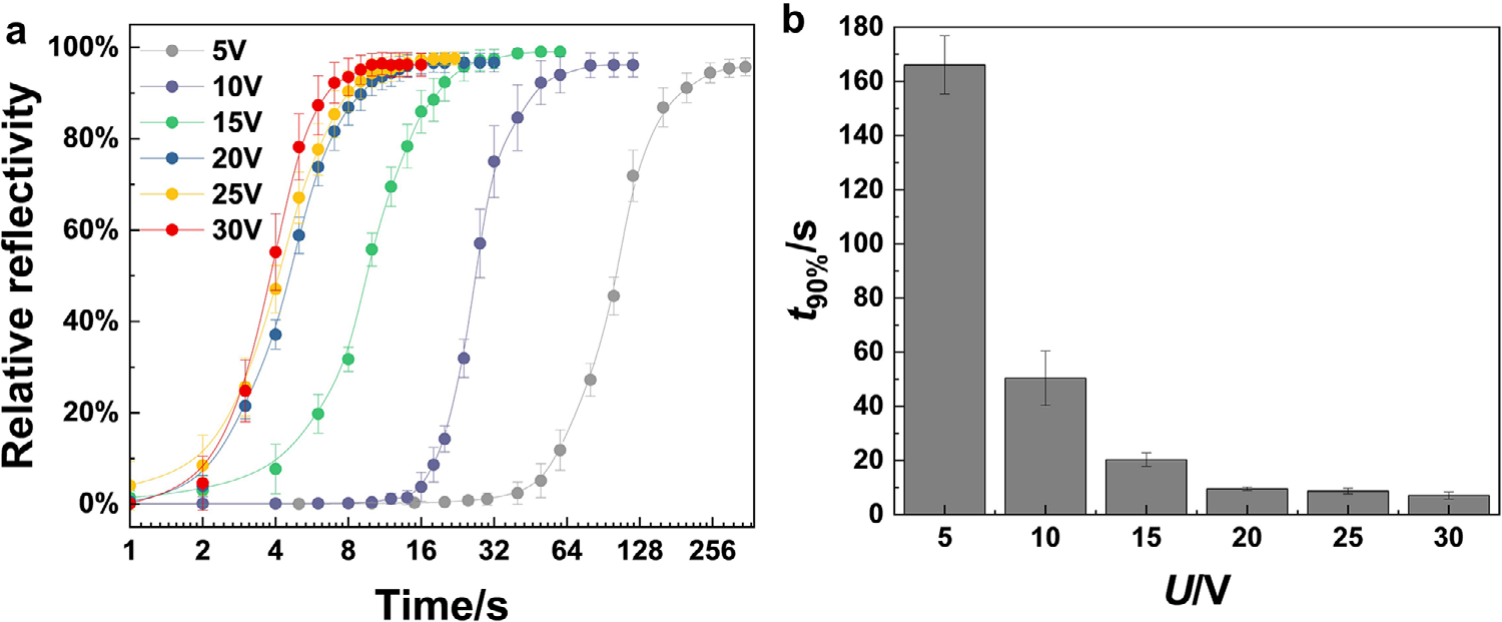
Effect of voltage on the plastron recovery efficiency of SHM‐E. (a) Variation of relative reflectivity with time during plastron self‐recovery under different voltages. (b) Variation of t90% (time to 90% relative reflectivity) as a function of voltage.

From an application perspective, higher voltages can enable faster recovery but inevitably increase energy consumption, and excessively rapid gas generation may promote bubble escape or excessive local bubble growth, which is less favorable for stable interfacial spreading. Considering that plastron loss in many underwater scenarios can be gradual, a low voltage (e.g., 5 V) may be sufficient for on‐demand replenishment and long‐term maintenance, whereas higher voltages may be useful for rapid recovery after strong disturbances.

### 2.4 Long‐Term Plastron Stability

To assess long‐term plastron maintenance of SHM‐E under static underwater conditions, we conducted a 16‐day continuous immersion test in tap water (conductivity: 189 ± 3 µS cm^−^^1^). For SHM (superhydrophobic mesh without electrolysis), the plastron gradually dissipated due to diffusion and related processes, and the coverage continuously decreased. As shown in Figure 5a, a pronounced decay occurred after 6 days of immersion, with the relative reflectivity dropping to ∼5%. The surface gradually changed from dark to bright, indicating near‐complete plastron loss and wetting transition (Figure 5b).

**FIGURE 5:**
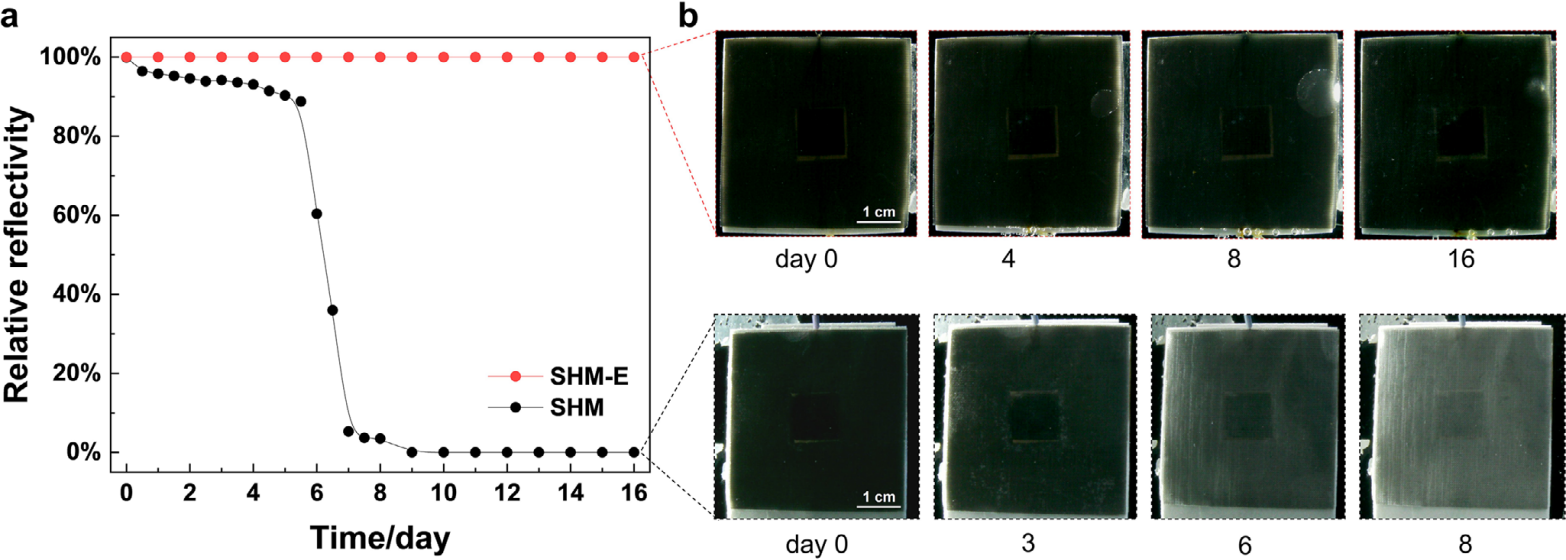
Long‐term plastron stability of SHM‐E. (a) Variation of plastron relative reflectivity with time for SHM (superhydrophobic mesh without electrolysis) and SHM‐E (5 V) in tap water. (b) Optical images of plastron evolution during the long‐term stability test: SHM‐E (pink dashed box) and SHM (black dashed box). After 16 days, SHM‐E maintains nearly complete plastron coverage, while SHM loses the entire plastron after 8 days.

In contrast, SHM‐E supplied under a constant applied voltage of 5 V exhibited much more stable plastron maintenance. Throughout the 16‐day period, the plastron coverage remained high, with relative reflectivity consistently above 95% and no obvious loss (Figure 5a,b). Periodic small bulged bubbles were observed on the surface (once every 2–3 days), suggesting an “on‐demand” working mode: minor plastron dissipation or local conduction caused by condensation/accumulation of moisture near the electrode could trigger weak electrolysis; once gas replenished the interface and re‐isolated the aqueous phase, the circuit disconnected automatically. During testing, the static current stayed below 1 mA and was typically only 300–800 µA. This low‐rate, continuous replenishment helps maintain high coverage without requiring strong electrolysis after large‐scale plastron failure. These results indicate that SHM‐E enables long‐term plastron maintenance and self‐regulated replenishment under low‐power operation.

### 2.5 Pressure Stability of the Plastron

To simulate hydrostatic pressure in deep‐water environments, we performed pressure tests from 0 to 200 kPa using a custom pressure setup (Figure S1) on SHM‐E and SHM. For SHM, the plastron gradually dissipated with increasing pressure, and the relative reflectivity continuously decreased, as shown in Figure 6a. When the pressure reached 40 kPa, the plastron was nearly completely lost and the surface appeared bright (Figure 6b).

**FIGURE 6:**
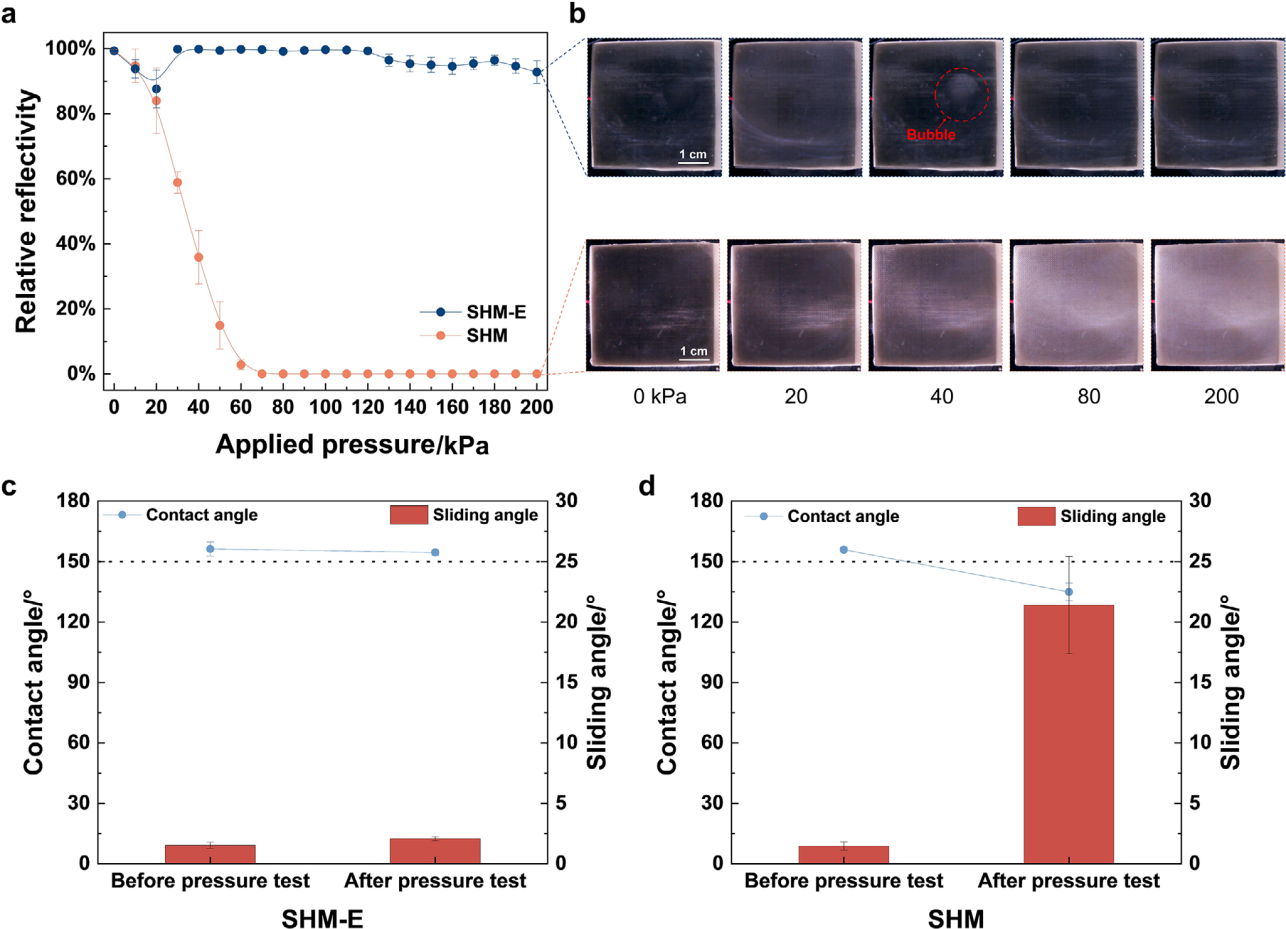
Underwater plastron pressure stability test. (a) Relative reflectivity evolution of submerged SHM and SHM‐E under 0–200 kPa hydrostatic pressure. (b) Corresponding optical profiles of SHM and SHM‐E during the pressure test. (c, d) Wettability changes of SHM and SHM‐E after the pressure test.

For SHM‐E, the pressure tolerance was markedly improved. At a hydrostatic pressure of 20 kPa, the relative reflectivity slightly decreased, similar to the trend observed in the non‐electrolysis SHM. At this stage, the plastron remained relatively intact, and the electrolysis circuit was not triggered. When the pressure increased further to 40 kPa, the relative reflectivity increased, indicating initiation of electrolysis and plastron self‐recovery. Bulged bubbles were observed as shown in Figure 6b, suggesting generation of excess gas. With continued pressurization up to 200 kPa, SHM‐E maintained self‐recovery upon plastron disturbance, and the relative reflectivity remained above 90%, indicating that the plastron stayed largely intact under high pressure. These results suggest that electrode integration enhances pressure stability and enables sustained plastron coverage under simulated deep‐water conditions.

During pressurization, electrolysis was activated only when pressure‐induced plastron damage caused sufficient water penetration to establish an ionic pathway between the electrodes. At approximately 40 kPa, the increase in relative reflectivity, together with the appearance of bulged bubbles, indicated the activation of electrolysis and the repair of the partially depleted plastron. This process differed from the complete removal–recovery process shown in Figure 2, as the plastron was only locally damaged under pressure. During further pressurization, analogous intermittent activation–termination events occurred only when the extent of plastron damage exceeded the threshold required for circuit conduction. Thus, increasing pressure did not result in continuous electrolysis; instead, sufficient local wetting activated gas generation, while subsequent gas replenishment displaced the infiltrated water and interrupted the conductive aqueous pathway.

To evaluate the effect of high pressure on wettability, both samples were held at 200 kPa for 30 min, removed, and immediately tested for contact angle (CA) and sliding angle (SA) as shown in Figure 6c,d. For SHM‐E, the CA changed only slightly from 156.2° ± 3.5° to 154.5° ± 1.4°, and the SA increased modestly from 1.5° ± 0.25° to 2.1° ± 0.16°, indicating minimal wettability degradation and retention of superhydrophobicity. In contrast, SHM exhibited a pronounced decrease in CA from 155.9° ± 1.2° to 134.9° ± 4.3°, and a substantial increase in SA from 1.5° ± 0.34° to 21.4° ± 4.0°, indicating loss of superhydrophobicity. This difference may be attributed to the absence of plastron protection in SHM under high pressure, which can promote liquid infiltration into microstructures or induce physical damage to the coating, thereby degrading superhydrophobicity. In SHM‐E, sustained plastron coverage can effectively isolate the coating from direct high‐pressure liquid contact, helping preserve wettability. Our previous studies demonstrated plastron recovery after pressure cycling [20], capillary‐assisted maintenance under flowing water [21], and gas‐film transport against buoyancy [48], supporting the ability of related mesh structures to retain, transport, and recover gas under the tested conditions.

### 2.6 Anti‐Scaling Performance

Underwater CaCO_3_ scaling typically involves ion accumulation, heterogeneous nucleation, and subsequent crystal growth/adhesion. For superhydrophobic surfaces, anti‐scaling behavior arises not only from reduced adhesion due to low surface energy, but more critically from the interfacial plastron in the Cassie state, which significantly reduces the effective solid–liquid contact area and thereby weakens CaCO_3_ nucleation and crystallization on the surface [49, 50, 51]. Previous studies have also indicated that scaling can intensify when the plastron becomes unstable or disappears during prolonged immersion, whereas surfaces that maintain a stable plastron can continuously suppress nucleation and deposition [52, 53]. Therefore, SHM‐E with on‐demand gas replenishment is expected to provide long‐term isolation of scaling ions through sustained plastron coverage and thus improve anti‐scaling reliability as shown in Figure 7a.

**FIGURE 7:**
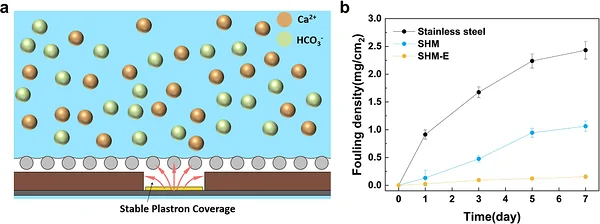
Anti‐scaling performance of SHM‐E. (a) Schematic of the anti‐scaling mechanism of SHM‐E. (b) Change in CaCO_3_ deposition mass on stainless steel substrates, SHM, and SHM‐E.

We evaluated stainless steel (SS), SHM, and SHM‐E in a scaling solution of 0.01 m CaCl_2_ and 0.01 m NaHCO_3_ at 60°C with stirring at 300 rpm for 7 days, and quantified CaCO_3_ deposition mass per unit area as a function of time (Figure 7b). Although deposition increased over time for all samples, the growth rates differed substantially. After 7 days, SS was almost fully covered by a dense scale layer (Figure S2), with a deposition mass of 2.43 mg cm^−^^2^. For SHM, as the plastron gradually deteriorated and the solid–liquid contact fraction increased, obvious scale adhesion occurred, yielding a deposition mass of 1.06 mg cm^−^^2^. In contrast, SHM‐E showed nearly no visible scale particles, with a deposition mass of only 0.15 mg cm^−^^2^, corresponding to reductions of 93.8% relative to SS and 85.8% relative to SHM.

Surface morphology and wettability after scaling further support the link between plastron stability and anti‐scaling performance. After scaling, SHM exhibited more pronounced scale coverage and severe degradation of superhydrophobicity, with droplet pinning observed (Figure S3). By contrast, SHM‐E retained typical superhydrophobic behavior after 7 days (Figure S4). Since SHM and SHM‐E share the same superhydrophobic coating and hierarchical structure (i.e., similar surface chemistry and roughness), the performance difference mainly originates from plastron lifetime: in SHM, plastron depletion allows Ca^2^^+^ and HCO_3_^−^ to accumulate near the substrate, promoting heterogeneous nucleation and continuous growth, leading to progressive scale buildup. In SHM‐E, the self‐responsive cycle (“plastron damage–circuit conduction–localized electrolysis–gas spreading repair–circuit disconnection”) enables continuous replenishment and self‐repair of the plastron, maintaining long‐term isolation of scaling ions from the substrate and suppressing nucleation and adhesion, thereby ensuring durable anti‐scaling performance.

## 3 Conclusion

We developed a structurally simple and low‐power underwater plastron self‐repair surface (SHM‐E) based on an integrated design combining a self‐spreading superhydrophobic mesh and locally integrated electrodes. This architecture addresses key limitations of existing electrolysis‐based gas replenishment approaches, including structural redundancy, gas loss, and the lack of self‐responsive operation. By coupling localized electrolysis with capillary‐assisted gas spreading, SHM‐E achieves global plastron restoration using a locally integrated Pt anode occupying only 1/16 of the surface area, eliminating the need for full‐area electrodes and external feedback control. The plastron itself serves as an intrinsic switch: wetting‐induced water ingress closes the circuit and initiates electrolysis, while gas replenishment re‐isolates the electrodes and autonomously terminates the current, enabling an unattended “damage–repair–dormancy” cycle. SHM‐E restores near‐complete plastron coverage within ≤60 s at 10 V, and maintains >95% coverage for 16 days at 5 V with a static current below 1 mA. The sustained gas layer improves pressure tolerance up to 200 kPa and preserves superhydrophobic wettability. Importantly, the stabilized plastron translates into robust anti‐scaling performance, reducing CaCO_3_ deposition by 93.8% relative to bare stainless steel. This strategy provides a practical design paradigm for durable plastron‐enabled interfaces relevant to drag reduction, pipeline anti‐scaling, and underwater device protection.

## 4 Experimental Section/Methods

### 4.1 Fabrication of SHM‐E Surfaces

The substrate and stainless‐steel mesh were sequentially ultrasonically cleaned in acetone, ethanol, and deionized water for 15 min each to remove oils and contaminants, followed by drying in an oven at 60°C for 2 h to ensure no residual moisture or impurities. The cleaned stainless‐steel mesh was then uniformly spray‐coated with a commercial superhydrophobic coating (NeverWet, Rust‐Oleum, USA) and dried at 60°C for 1 h to obtain a superhydrophobic mesh. For assembly, a Pt plate electrode (1 cm × 1 cm × 0.2 mm) was fixed at the center of the substrate using double‐sided tape as the anode. Double‐sided foam tape (thickness: 1 mm) was attached to the non‐anode area to form a hollow spacer surrounding the Pt plate, creating a central cavity with dimensions of 1 cm × 1 cm and a depth of 0.8 mm. The superhydrophobic stainless‐steel mesh was placed on top of the foam spacer as the cathode and pressed to ensure intimate contact, forming a sealed cavity. The cathode was connected to the negative terminal of a DC power supply, and the anode was connected to the positive terminal, yielding the SHM‐E surface.

### 4.2 Characterization

Contact and sliding angles were measured at room temperature using a video‐based optical angle measurement system (OCA25) with 5 µL deionized‐water droplets. For each condition, five independent droplets were tested on each of three square specimens (4 cm × 4 cm), giving a total of 15 measurements (n = 15). Surface morphology was examined by environmental scanning electron microscopy (ESEM; FEI Quanta FEG 200).

### 4.3 Characterization of Plastron Underwater

An optical observation setup was built for characterizing the underwater plastron. Specimens were placed horizontally in an acrylic water chamber filled with tap water (conductivity: 189 ± 3 µS cm^−^^1^, measured at 25°C) and illuminated by a directional LED cold light source at a low incident angle (∼10°). Owing to total internal reflection at the gas–water interface, the presence/absence of the plastron produced an evident brightness contrast, which was continuously recorded by a high‐resolution camera mounted perpendicularly above the sample. The acquired images were processed in image analysis software ImageJ via grayscale conversion and binary thresholding to distinguish plastron‐covered and wetted regions. The fraction of dark pixels was defined as the relative reflectivity (RR, 0%–100%), calculated as the ratio of dark pixel count to the total pixel count (normalized for each run), and was used to quantify plastron coverage. The temporal evolution of the plastron was then obtained by plotting RR as a function of elapsed time.

### 4.4 Plastron Self‐Recovery and Stability Tests

Macroscopic plastron removal was simulated by gently aspirating and displacing the optically observable gas layer retained within the mesh openings and central cavity using a pipette. The resulting local pressure disturbance removed the continuous micrometer‐scale plastron and allowed water to enter the larger mesh–cavity structure, but was not intended to force complete wetting of every nanoscale cavity within the nanoparticle coating. A camera (30 fps) recorded the entire “removal–recovery” process. SHM‐E was connected to a constant‐voltage power supply (5, 10, 15, 20, 25, and 30 V), and the circuit current was monitored in real time using a multimeter. The correlation between plastron coverage (relative reflectivity) and current evolution was analyzed.

For long‐term immersion stability, samples were placed in tap water at a depth of ∼5 cm. An electrolysis group (5 V constant‐voltage power supply) and a non‐electrolysis group were tested for 16 days, with periodic recording of plastron coverage and surface appearance.

For pressure stability, samples were placed in a water reservoir (water depth: 2 cm) inside a self‐built pressure chamber (Figure S1). The chamber consisted of a stainless‐steel cylindrical vessel (inner diameter 133 mm, height 100 mm) rated for 1 MPa, equipped with a precision pressure regulator and manometer, and a glass viewport for camera monitoring. The vessel was pressurized with high‐pressure gas from 0 to 200 kPa, increasing by 10 kPa every 20 s to simulate deep‐water hydrostatic pressure. Both an electrolysis group (10 V constant‐voltage power supply) and a non‐electrolysis group were tested. After reaching 200 kPa, the pressure was held for 30 min. Samples were then removed, and their wettability was evaluated by measuring contact angle and sliding angle before and after the test.

### 4.5 Anti‐Scaling Test

Three types of samples—SHM‐E (5 V constant‐voltage power supply), SHM (superhydrophobic mesh without electrolysis), and stainless‐steel substrate—were prepared with identical dimensions (4 cm × 4 cm). Samples were immersed in 500 mL of a scaling solution containing 0.01 m CaCl_2_ and 0.01 m NaHCO_3_. The test was conducted at 60°C with magnetic stirring at 300 rpm to ensure solution homogeneity and mass transfer. During the test, sample mass changes were periodically measured to determine the CaCO_3_ deposition mass per unit area as a function of time. The total test duration was 7 days to simulate scaling during long‐term operation.

## Author Contributions

**Jiaming Wang**: conceptualization, methodology, Writing – original draft, Writing – review and editing, formal analysis, investigation, data curation. **Zhen Zhang**: writing – review and editing, validation. **Yuhong Liu**: writing – review and editing, project administration, resources, funding acquisition, supervision.

## Conflicts of Interest

The authors declare no conflicts of interest.

## Supporting information




**Supporting File**: advs78069‐sup‐0001‐SuppMat.docx.

## Data Availability

The data that support the findings of this study are available from the corresponding author upon reasonable request.
