# Supplementary material for: Self‐Responsive Plastron Maintenance on a Superhydrophobic Mesh via Locally Integrated Electrolysis
Source: Adv Sci (Weinh). 2026 Oct 8:e78069. Online ahead of print. doi: 10.1002/advs.78069 (PMC13650025; doi:10.1002/advs.78069)
Supplement: Supplementary file 1 [file ADVS-9999-e78069-s001.docx]

Supporting Information

Self-Responsive Plastron Maintenance on a Superhydrophobic Mesh via Locally Integrated Electrolysis

Jiaming Wang, Zhen Zhang, Yuhong Liu*

**Figure S1.** Photograph of the custom-built pressure chamber used for plastron stability tests. The setup consists of a stainless-steel cylindrical vessel (inner diameter: 133 mm; height: 100 mm; maximum working pressure: 1 MPa) fitted with a precision pressure regulator and a manometer. Samples were immersed in tap water inside the vessel, and the internal pressure was increased stepwise by introducing compressed air from an air pump. Plastron evolution was monitored through the observation window by recording the relative reflectivity with an optical camera.

**Figure S2. Fouling behavior of the stainless steel substrate.** (a) Uncontaminated stainless steel substrate. (b) Stainless steel substrate after 7 days of fouling.

**Figure S3. Fouling behavior of the SHM.** (a) SHM surface after 7 days of fouling and drying, with local scale deposits marked by the red dashed circle. (b) After dropping a water droplet on the SHM, wetting pinning occurs

**Figure S4. Fouling behavior of the SHM-E.** (a) SHM-E surface after 7 days of fouling and drying, with almost no visible scale deposits. (b) After dropping a water droplet on the SHM-E, the liquid maintains a spherical shape, and the surface still retains superhydrophobicity

**Estimation of gas transport flow rate driven by capillarity on SHM**

The thickness of the plastron of SHM-E is about:

$D_{p}=2D=126 \mu m$	(S1)

The plastron volume fraction in the mesh architecture

$S_{p}=\frac{V_{t\mathrm{otal}}-V_{\mathrm{mesh}}}{V_{\mathrm{total}}}=\frac{2D{(W+D)}^{2}-4*\frac{1}{2}*\frac{1}{4}\pi D^{2}\sqrt{{(W+D)}^{2}+D^{2}}}{2D{(W+D)}^{2}}=69.6\%$	(S2)

For a 4 cm × 4 cm SHM-E sample, the total plastron volume equals the sum of the plastron volume retained within the mesh structure and that within the air cavity

$V_{p}=4 cm*4 cm*D_{p}*S_{p}+0.8 mm*1 cm*1 cm=0.22 ml$	(S3)

Assuming that the plastron has been fully restored at 50 s, the electrolysis-driven gas generation rate can be estimated as

$Q_{E}=\frac{V_{p}}{50s}=4.4 \mu l/s$	(S4)
